# Comparative analysis of hemostatic efficacy and safety: absorbable gelatin sponge with hemostatic fluid gelatin vs. tranexamic acid in posterior cervical expansive open-door laminoplasty

**DOI:** 10.3389/fsurg.2025.1668904

**Published:** 2025-09-26

**Authors:** Yu Yang, Donghui Cao, Xusheng Li, Xiao Zhang, Yanrong Tian, Pengcheng Mao, Haifeng Yuan

**Affiliations:** 1 Department of Spinal Orthopedics, General Hospital of Ningxia Medical University, Yinchuan, China; 2 First Clinical Medical College, Ningxia Medical University, Yinchuan, China

**Keywords:** absorbable gelatin sponge, hemostatic fluid gelatin, tranexamic acid, hemostatic material, posterior cervical expansive open-door laminoplasty

## Abstract

**Background:**

Posterior cervical expansive open-door laminoplasty carries significant intraoperative bleeding risks from Batson's plexus. While hemostatic agents such as absorbable gelatin sponge (GS), hemostatic fluid gelatin (HFG), and tranexamic acid (TXA) are widely used in spinal surgery, the efficacy and safety of combined strategies remain underexplored. This study aims to compare the hemostatic efficacy and safety of GS combined with HFG vs. GS combined with intravenous TXA in posterior cervical expansive open-door laminoplasty.

**Methods:**

A retrospective analysis was conducted on 75 patients undergoing posterior cervical expansive open-door laminoplasty at Ningxia Medical University General Hospital from January 2022 to January 2024, with stratification into two groups based on intraoperative hemostatic materials: the GS-HFG group (absorbable gelatin sponge combined with hemostatic fluid gelatin, *n* = 30) and the GS-TXA group (absorbable gelatin sponge combined with intravenous tranexamic acid, *n* = 45), with subsequent comparison of baseline demographics, operative duration, intraoperative blood loss, total postoperative drainage volume, Visual Analog Scale (VAS) pain scores, Japanese Orthopaedic Association (JOA) functional assessments, postoperative adverse events, and complications.

**Results:**

Operative duration demonstrated no statistically significant difference between the GS-HFG and GS-TXA groups (139.9 ± 21.7 min vs. 144.4 ± 30.7 min; *p* > 0.05), with intraoperative blood loss measuring 135 ± 29.1 ml vs. 145 ± 29.4 ml (mean difference: −10 ml; 95% CI: 134.9–144.7 ml; *p* > 0.05), and postoperative drainage volumes recorded at 200.65 ± 48.92 ml vs. 186.24 ± 52.18 ml (*p* > 0.05). No significant differences were observed in preoperative/postoperative biochemical markers. Complications included axial symptoms (1 case in GS-HFG, 3 in GS-TXA) and C5 nerve root palsy (1 case in each group). No thromboembolic events or other adverse reactions occurred.

**Conclusion:**

Both the GS-HFG and GS-TXA protocols effectively reduce perioperative bleeding without increasing the risk of complications in posterior cervical expansive open-door laminoplasty.

## Introduction

The posterior cervical expansive open-door laminoplasty was first proposed by the Japanese scholar Hirabayashi in 1977 (1). It is currently an effective surgical method for treating multilevel cervical spondylotic myelopathy (CSM), as well as developmental or degenerative spinal canal stenosis, and ossification of the posterior longitudinal ligament (OPLL) (2). However, this procedure necessitates extensive soft tissue dissection in proximity to the epidural venous plexus (Batson's plexus). The plexus is highly susceptible to injury during lamina elevation, predisposing to increased intraoperative blood loss (IBL), which may trigger severe complications including hemodynamic instability, organ hypoxia, spinal cord ischemia, electrolyte imbalances, and hemorrhagic shock (3, 4). Consequently, effective management of intraoperative and perioperative haemorrhage constitutes a critical determinant for ensuring procedural safety and achieving a favourable patient prognosis.

Currently, various hemostatic agents, including absorbable gelatin sponge (GS), hemostatic fluid gelatin (HFG), and tranexamic acid (TXA), are widely utilised in spinal surgery. GS, primarily composed of porcine-derived gelatin, achieves hemostasis by absorbing multiple times its weight in blood, which concentrates clotting factors and initiates localised coagulation. It is particularly indicated for controlling low-pressure oozing at sites where bone wax application is contraindicated or electrocautery carries unacceptable neural risks (5, 6). Compared with GS, HFG provides a physical scaffold for platelets, enabling them to coagulate into blood clots. Its flowing form not only reaches deep cavities that are difficult for GS to access, but also does not compress the cauda equina or spinal cord (7). TXA, a synthetic antifibrinolytic agent, competitively inhibits plasminogen activation and fibrin degradation. Its systemic hemostatic efficacy in reducing perioperative blood loss has been validated in cardiac and spinal surgeries (8, 9). Studies have shown that these three hemostatic materials exhibit good hemostatic efficacy and safety in orthopaedic surgeries (10–12).

Although individual hemostatic materials are widely used, there are limited reports on the efficacy and safety of combined hemostatic strategies in posterior cervical open-door laminoplasty. Therefore, this study retrospectively analysed patients with CSM who underwent posterior cervical open-door laminoplasty at the General Hospital of Ningxia Medical University from January 2022 to January 2024, aiming to investigate the hemostatic efficacy and safety of the GS-HFG group vs. the GS-TXA group in this procedure.

## Materials and methods

### Clinical data

This study retrospectively analysed the data of 75 patients who underwent posterior cervical open-door laminoplasty at the General Hospital of Ningxia Medical University from January 2022 to January 2024. According to the different intraoperative hemostatic materials used, patients were divided into two groups: the GS-HFG group (*n* = 30, 13 males and 17 females, age 65.2 ± 7.5 years) and the GS-TXA group (*n* = 45, 19 males and 26 females, age 58.5 ± 8.4 years). There were no statistically significant differences in gender, age, or other baseline characteristics between the two groups (*P* > 0.05) (Table 1). The study was approved by the Ethics Committee of the General Hospital of Ningxia Medical University (Approval No.: KYLL-2025-1356), and written informed consent was obtained from all patients.

**Table 1:** Baseline characteristics of patients.

Parameter	GS-HFG group (*n* = 30)	GS-TXA group (*n* = 45)	Statistical value	*P* value*
Age (years)	60.8 ± 8.7	59.6 ± 7.9	*t* = 3.615	0.417
Sex (male/female)	13/17	19/26	*χ*^2^ = 0.009	0.924
Weight (kg)	64.4 ± 8.4	66.7 ± 8.6	*t* = −1.132	0.262
Smoking (yes/no)	13/17	14/31	χ^2^ = 1.167	0.280
Hypertension (yes/no)	12/18	18/27	*χ^2^* = 0.000	1.000
Diabetes (yes/no)	12/18	9/36	*χ*^2^ = 3.571	0.058
ASA classification				0.326
II	7	17	–	–
III	20	26	–	–
IV	3	2	–	–

* The unpaired *t*-test was used for the analysis of age, weight.

The chi-squared test for sex, smoking, hypertension, diabetes and ASA classification.

### Inclusion and exclusion criteria

The inclusion criteria were: (1) Multilevel CSM, stenosis, or OPLL; (2) aged 18–85 years; (3) failed standard conservative treatment and required posterior cervical open-door laminoplasty; (4) Clear surgical indications.

The exclusion criteria were: (1) Previous history of cervical spine surgery; (2) Comorbid bleeding disorders or other severe systemic diseases affecting coagulation function; (3) Intraoperative or postoperative cerebrospinal fluid leakage (CSF leakage); (4) Allergy to GS, HFG, or TXA; (5) Cervical spine trauma, tumors, infections, or congenital malformations.

## Therapeutic methods

### Surgical procedure and postoperative management

All surgeries were performed by the same senior attending surgeon. Under general anaesthesia, patients were placed in the prone position. Standard posterior cervical open-door laminoplasty was performed in all cases (1), involving careful elevation of the lamina followed by the selection and fixation of five Johnson & Johnson mini-plates: two 6 mm titanium screws secured the lateral mass end to the lateral mass, and an 8 mm screw fixed the lamina end to the lamina. After ensuring no active bleeding, the incision was closed, and two drainage tubes were routinely placed adjacent to the incision.

Postoperatively, all patients received anti-inflammatory and decongestant/analgesic therapy. Drainage tubes were removed when output was <50 ml. Patients were instructed to ambulate with cervical collar protection within 4 weeks after surgery.

### Hemostatic technique for GS-HFG group

During the surgical procedure, after completion of the laminectomy and upon identification of significant bleeding points, absorbable gelatin sponge (trade name: Absorbable Gelatin Sponge; Xiangen Medical, China) was cut to the appropriate size and applied with gentle pressure to the bleeding sites for initial hemostasis through physical occlusion. Subsequently, the expanded GS was removed, and pre-mixed absorbable hemostatic fluid gelatin (trade name: Surgiflo; Johnson & Johnson, USA) was uniformly applied over the gelatin sponge surface and surrounding bleeding tissues. Fresh GS was then layered over the HFG to augment hemostatic efficacy.

### Hemostatic technique for GS-TXA group

Ten minutes before skin incision, patients received an intravenous infusion of tranexamic acid (1 g TXA injection mixed in 100 ml of 0.9% sodium chloride solution) at a rate of 30 drops/min. TXA reduced systemic blood loss by inhibiting the fibrinolytic system. During the operation, hemorrhagic sites encountered were managed with GS for localised compression hemostasis, leveraging complementary mechanisms to reduce intraoperative blood loss and optimise surgical conditions.

### Outcome measures

The following parameters were compared between the two groups: operative time, intraoperative blood loss, total postoperative drainage volume, duration of postoperative drainage, length of postoperative hospital stay, and levels of hemoglobin (HB), hematocrit (HCT), hepatic function, renal function, and coagulation profiles measured preoperatively and on postoperative day 1 (POD1). Additionally, Visual Analogue Scale (VAS) and Japanese Orthopaedic Association (JOA) scores were assessed before and after surgery, along with documentation of complications and adverse events.

### Statistical analysis

Statistical analyses were performed using SPSS software (version 26.0). Quantitative data, including operative time, intraoperative blood loss, and postoperative drainage volume, were expressed as mean ± standard deviation (SD). Intergroup comparisons were conducted using an independent samples *t*-test, while intragroup comparisons employed one-way analysis of variance (ANOVA). For data violating normality assumptions, the nonparametric Mann–Whitney *U* test was applied. Categorical data comparisons utilised the chi-square (*χ*^2^) test. A *P* value < 0.05 was considered statistically significant.

## Results

### Baseline characteristics

**Figure 1:**
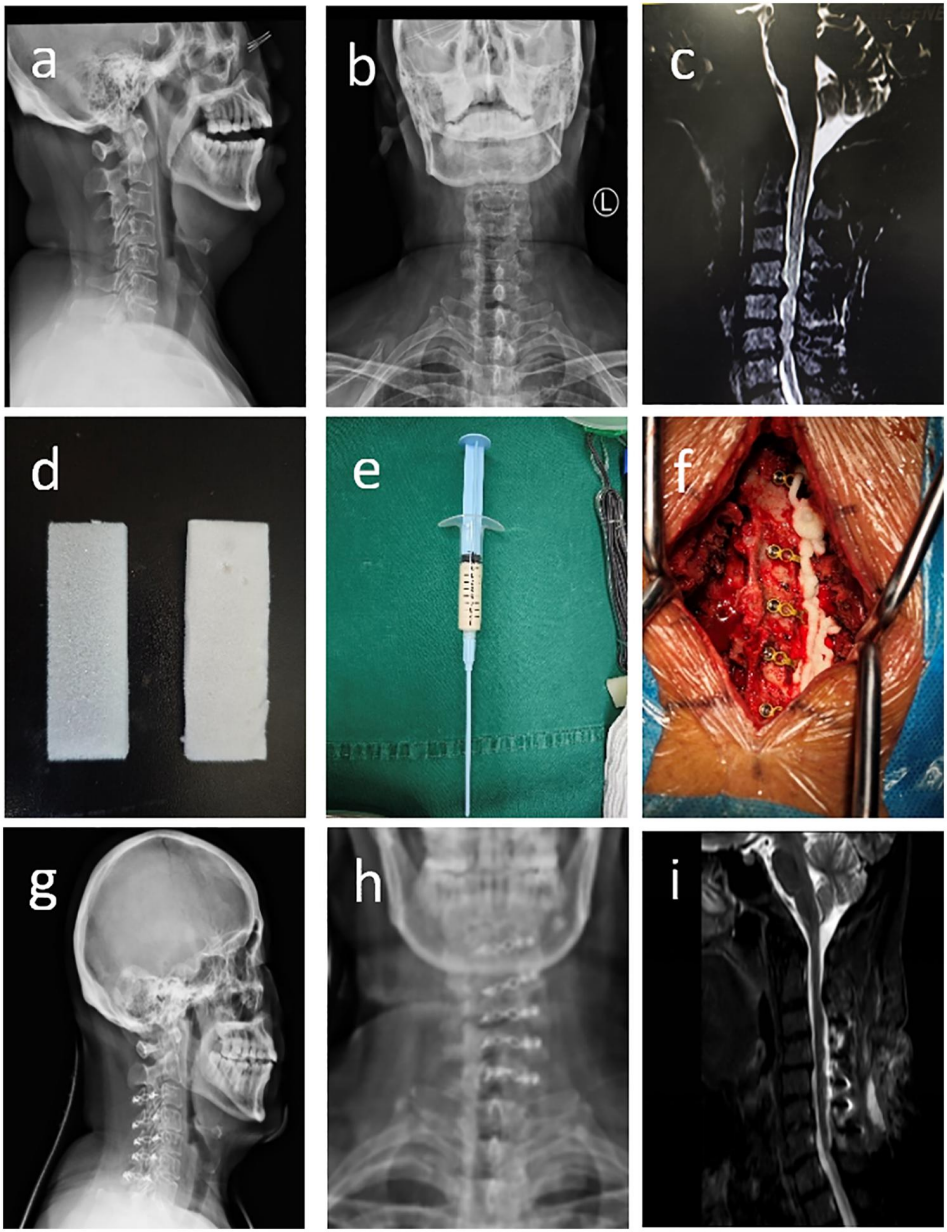
**(a–c)** Preoperative cervical spine radiographs (anteroposterior and lateral views) and MRI demonstrating C4-C7 disc herniations with spinal cord compressed and deformed; **(d–f)** absorbable gelatin sponge (GS), pre-mixed flowable gelatin hemostatic agent (HFG), and intraoperative application of flowable gelatin for hemostasis; **(g–i**) postoperative cervical spine radiographs (anteroposterior and lateral views) showing well-positioned internal fixation, and MRI demonstrating decreased spinal cord compression.

No statistically significant differences were observed in gender, age, or other baseline characteristics between the two groups (*P* > 0.05) (Table 1). All patients successfully underwent posterior cervical expansive open-door laminoplasty. Follow-up duration exceeded 6 months for all participants. Representative cases are shown in Figure 1.

### Intraoperative outcomes

Calculated intraoperative blood loss (IBL), determined using the Gross formula based on demographic data and preoperative/postoperative day 1 hematocrit (HCT) (13), served as the primary outcome. Mean IBL was 135 ± 29.1 ml in the GS-HFG group vs. 145 ± 29.4 ml in the GS-TXA group (mean difference: −10 ml; 95% CI, 134.9–144.7 ml; *P* > 0.05). No significant intergroup difference in IBL was observed (Table 2). Operative time was 139.9 ± 21.7 min for GS-HFG and 144.4 ± 30.7 min for GS-TXA (*P* > 0.05), with no statistically significant difference (Table 3).

**Table 2:** Primary outcome measures.

Parameter	GS-HFG group (*n* = 30)	GS-TXA group (*n* = 45)	Statistical value	*P* value*
Intraoperative blood loss (ml)	135.0 ± 29.1	145.0 ± 29.4	*t* = −1.419	0.160
Preoperative haemoglobin (g/L)	140.0 ± 18.3	139.0 ± 14.3	*t* = 0.272	0.790
Postoperative haemoglobin (g/L)	128.0 ± 13.5	125.0 ± 14.2	*t* = 0.810	0.420
Preoperative hematocrit (%)	41.5.0 ± 4.0	41.7 ± 2.7	*t* = −0.258	0.815
Postoperative hematocrit (%)	38.6 ± 3.4	38.2 ± 2.8	*t* = 0.475	0.64

* The independent samples *t*-test was used for the analysis of intraoperative blood loss, preoperative haemoglobin, postoperative haemoglobin, preoperative hematocrit and postoperative hematocrit.

**Table 3:** Intraoperative and postoperative outcomes.

Parameter	GS-HFG group (*n* = 30)	GS-TXA group (*n* = 45)	Statistical value	*P* value*
Operative time (min)	139.9 ± 21.7	144.4 ± 30.7	*t* = −0.69	0.490
Intraoperative transfusion (ml)	0	0	0	0
Total postoperative drainage volume (ml)	200.6 ± 48.9	186.2 ± 52.1	*t* = 1.525	0.214
Drainage duration (days)	3.6 ± 0.97	3.23 ± 0.73	*t* = 1.327	0.815
Postoperative hospitalisation (days)	4.23 ± 0.57	3.83 ± 0.96	*t* = 1.835	0.927

* The independent samples *t*-test was used for the analysis of operative time, total postoperative drainage volume, drainage duration and postoperative hospitalization.

### Postoperative outcomes

Total postoperative drainage volume was 200.65 ± 48.92 ml in the GS-HFG group compared to 186.24 ± 52.18 ml in the GS-TXA group (*P* > 0.05), indicating no significant difference. Other postoperative parameters, including drainage duration and length of hospital stay, also showed no significant intergroup differences (*P* > 0.05) (Table 3).

### Biochemical parameters

**Figure 2:**
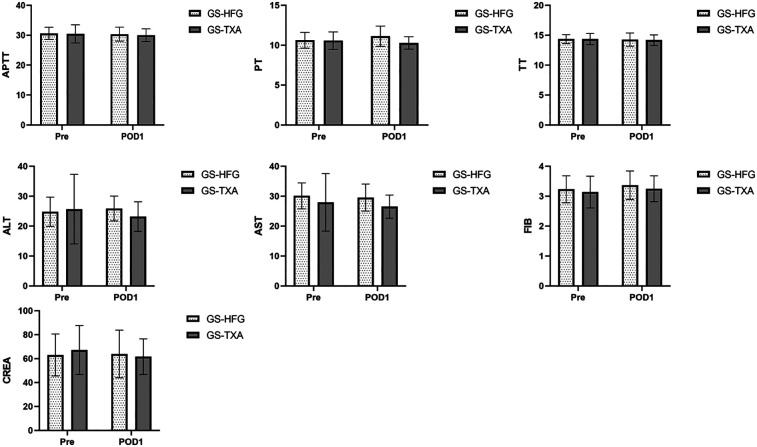
The APTT, PT, FIB, TT, AST, ALT and CREA in the GS-HFG and GS-TXA groups. AST, aspartate aminotransferase; ALT, alanine aminotransferase; CREA, creatinine; APTT, activated partial thromboplastin time; PT, prothrombin time; TT, thrombin time; FIB, fibrinogen; Pre, preoperative; POD1, postoperative day 1.

No significant perioperative changes were detected in aspartate aminotransferase (AST), alanine aminotransferase (ALT), creatinine (CREA), activated partial thromboplastin time (APTT), prothrombin time (PT), thrombin time (TT), or fibrinogen (FIB) levels. Intergroup comparisons of pre- and postoperative day 1 data revealed no statistically significant differences (*P* > 0.05) (Figure 2).

### VAS and JOA scores

**Figure 3:**
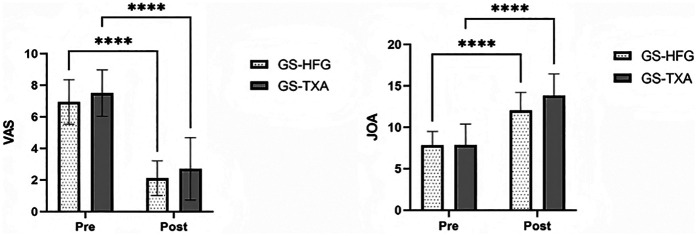
JOA and VAS scores for arm and neck pain preoperatively and at discharge.

Postoperatively, both groups demonstrated statistically significant improvements in VAS and JOA scores compared to preoperative assessments (*P* < 0.05) (Figure 3).

### Postoperative complications and adverse events

In the GS-HFG group, axial symptoms developed in 1 patient, and C5 nerve root palsy occurred in 1 patient. The GS-TXA group exhibited 3 cases of axial symptoms and 1 case of C5 nerve root palsy. No thromboembolic complications (including deep vein thrombosis or pulmonary embolism) or other adverse events were documented in the remaining patients during the postoperative period.

## Discussion

Posterior cervical expansive open-door laminoplasty, as the preferred surgical procedure for multilevel cervical spondylotic myelopathy, still faces the key clinical challenge of intraoperative haemorrhage control (14). Since the Batson venous plexus on the dorsal aspect of the vertebral body surrounds the dural sac, lamina open-door manipulation easily causes difficult-to-control haemorrhage; coupled with venous return disorder caused by long-term compression from compressive structures (intervertebral discs, osteophytes, etc.), this further increases bleeding risk during decompression (15). Traditional bipolar electrocautery has a limited hemostatic effect on epidural microvessels and may cause nerve injury. Therefore, rational application of hemostatic materials is crucial for controlling perioperative blood loss. Although GS, HFG, and TXA have been widely used in orthopaedic surgery with proven effectiveness (6, 11, 16), there are few reports on the hemostatic efficacy and safety of combined hemostatic strategies in cervical spine surgery. This study investigates the hemostatic effects and safety of two combined schemes, GS-HFG and GS-TXA, in cervical expansive open-door laminoplasty.

In cervical posterior expansive open-door laminoplasty, the two combined hemostatic schemes exhibit distinct mechanisms of action. When used alone, GS is typically trimmed and packed into the epidural space for physical compression hemostasis. However, its significant water-absorbing expansion property carries potential risks of neural and spinal cord compression. Friedma et al. (17) and Alander et al. (18) separately reported one case of quadriplegia caused by GS expansion after lumbar surgery and one after anterior cervical surgery, respectively. In contrast, HFG utilises its excellent fluidity to uniformly cover bleeding surfaces, forming a biological film that accelerates coagulation (19). Nevertheless, when intraspinal bleeding occurs rapidly, HFG tends to be dispersed by blood flow. When GS and HFG are combined, GS provides physical scaffolding, and HFG penetrates micro-bleeding sites via its fluidity, thereby enhancing hemostatic efficacy synergistically. TXA, administered primarily intravenously, systemically inhibits plasminogen activation and reduces fibrin degradation, thereby holistically decreasing the bleeding tendency (20, 21). With GS-TXA, local physical hemostasis is combined with systemic antifibrinolysis; however, TXA's efficacy remains limited for non-hyperfibrinolytic haemorrhage (22).

Our study revealed no statistically significant difference in intraoperative blood loss between the GS-HFG and GS-TXA groups during cervical posterior expansive open-door laminoplasty, indicating comparable effectiveness of both combined hemostatic regimens for controlling surgical haemorrhage. However, slightly reduced intraoperative bleeding was observed in the GS-HFG cohort, attributable to HFG's superior fluidity, which enables the rapid formation of stable hemostatic barriers, particularly effective for microvascular and dispersed bleeding sites. In contrast, TXA provides systemic fibrinolytic suppression, which may result in slower hemostatic speed for active bleeding compared to HFG. Conversely, the GS-TXA group exhibited significantly lower postoperative drainage volume than the GS-HFG group, suggesting TXA's potential advantage in reducing hidden blood loss during the postoperative phase, as indicated by Sun et al. (23) demonstrating intravenous TXA effectively decreases perioperative total and hidden blood loss in posterior lumbar interbody fusion (PLIF), with Abdou et al.'s meta-analysis (24) further confirming that low-dose TXA minimally affects intraoperative blood loss or transfusion requirements but significantly reduces postoperative bleeding.

Our study found no statistically significant differences between the two groups in postoperative hospital stay or drainage days, indicating that both hemostatic regimens had no significant impact on postoperative recovery. However, the GS-TXA group showed slightly shorter postoperative hospital stay and lower drainage volume than the GS-HFG group. This may imply potential clinical benefits, including a reduced risk of infection and lower hospitalisation costs. Postoperatively, only a small proportion of patients in both groups developed axial symptoms and C5 nerve root palsy, with no serious adverse events observed, consistent with previous studies (7, 25, 26). This safety demonstration is directly attributed to the biocompatibility of hemostatic agents and the precision of their surgical application in both groups. Furthermore, no significant changes were found in postoperative biochemical indicators, further confirming the safety of both combined regimens in the study population. Zhang et al. observed in scoliosis surgery that when blood loss reached 800 ml, coagulation indices such as APTT and PT showed no significant fluctuations, which may be attributed to the compensatory capacity of the coagulation system to maintain homeostasis (27).

This study has several limitations: it is a retrospective single-center study, and thus the conclusions should be further validated by prospective randomized controlled trials; dynamic fibrinolysis indicators such as D-dimer peak levels were not monitored, preventing quantification of TXA's inhibitory effect on the fibrinolytic system; the sample size was relatively small; and the follow-up duration was insufficient. Future studies will continue to expand the sample size, extend the follow-up period, and incorporate additional research indicators, with the aim of obtaining more robust scientific findings. In addition, we did not evaluate the potential clinical value of using all three hemostatic agents (GS, HFG, and TXA) together, due to the lack of relevant cases. The actual benefits, risks, and cost-effectiveness of the triple combination remain unclear, and future studies are needed to address this issue.

## Conclusion

Our results demonstrate that both the GS-HFG group and GS-TXA group effectively controlled perioperative bleeding with favourable safety profiles during posterior cervical expansive open-door laminoplasty. The GS-HFG group leveraged its fluid properties and physical sealing capability to demonstrate superior efficacy in controlling microvascular bleeding from the epidural venous plexus intraoperatively. Conversely, the GS-TXA group demonstrated a comparative advantage in reducing postoperative hidden blood loss due to its systemic antifibrinolytic effect. Clinicians may select the more appropriate combined hemostatic regimen based on individual patient characteristics and bleeding patterns.

## Data Availability

The raw data supporting the conclusions of this article will be made available by the authors, without undue reservation.
